# Pt-Sn alloy shells with tunable composition and structure on Au nanoparticles for boosting ethanol oxidation

**DOI:** 10.3389/fchem.2022.993894

**Published:** 2022-08-30

**Authors:** Ningkang Qian, Liang Ji, Xiao Li, Jingbo Huang, Junjie Li, Xingqiao Wu, Deren Yang, Hui Zhang

**Affiliations:** ^1^ State Key Laboratory of Silicon Materials and School of Materials Science and Engineering, Zhejiang University, Hangzhou, Zhejiang, China; ^2^ Institute for Carbon Neutralization, College of Chemistry and Materials Engineering, Wenzhou University, Wenzhou, Zhejiang, China; ^3^ Zhejiang Provincial Key Laboratory of Power Semiconductor Materials and Devices, ZJU-Hangzhou Global Scientific and Technological Innovation Center, Hangzhou, Zhejiang, China

**Keywords:** core−shell, AuPtSn, tunable composition and structure, trimetallic, nanoparticles, ethanol oxidation

## Abstract

Combining the core-shell structure with the optimization of surface composition and structure in the shell is a fantastic strategy to enhance the electrocatalytic performances. Here, we synthesized trimetallic Au@Pt_x_Sn_y_ core-shell nanoparticles (NPs) with tunable composition and structure of Pt-Sn alloyed shells. Impressively, the Au@PtSn core-shell NPs with hexagonal PtSn alloyed shells exhibited the highest mass activity and specific activity toward ethanol oxidation reaction (EOR) in alkaline electrolyte, which are 13.0 and 12.7 times higher than those of the commercial Pt/C. In addition, the Au@PtSn core-shell NPs displayed the best stability compared to commercial Pt/C, with only 44.8% loss vs. 86.8% loss in mass activity after 1,000 s due to the stronger anti-poisoning ability for reaction intermediates. The theory calculations reveal that the introduction of Au core and alloying Pt with Sn both endow Pt with an appropriate d-band center, and thus effectively boosting the EOR activity.

## Introduction

Direct ethanol fuel cells (DEFCs) have emerged as a competitive candidate among the energy conversion devices. Ethanol, as a renewable source of energy, not only possesses high energy density, but also can be derived in large quantities from biomass. Furthermore, ethanol takes the advantages of ease of storage and transportation of liquid fuels, avoiding some thorny problems caused by gaseous fuels (e.g., H_2_) (Kamarudin et al., 2013; Akhairi and Kamarudin, 2016). Platinum (Pt) and Pt-based nanomaterials are considered to be the most promising catalysts towards the ethanol oxidation reaction (EOR) at the anode (Kowal et al., 2009; Li et al., 2010; Sulaiman et al., 2017; Li M. et al., 2019). However, Pt-based catalysts suffers from scarce abundance and high price of Pt, and is prone to be poisoned by the byproducts (e.g., CO) generated during reaction (Li J. et al., 2019; Wang et al., 2019; Luo et al., 2020; Yang et al., 2021), leading to low efficiency in activity and poor durability. Therefore, it is urgent to enhance the performance of electrocatalysts for EOR and simultaneously reduce the usage of Pt through rational design of compositions and structures.

Production of core-shell structure is an effective strategy to increase the atomic utilization of shell metal in nanocrystals. Meanwhile, the interactions between the core and shell metals may improve the electrocatalytic performances (Li et al., 2020; He et al., 2021b; Wang et al., 2021; Wu et al., 2021). As reported previously, the tensile strain on the Pt-skin introduced by the inner cores, such as Au, would be benefit to boost the activity for EOR (Li et al., 2012; Liang et al., 2019; Zhang et al., 2019; Luo et al., 2021). Another promising route is alloying Pt with a second metal, such as Rh (Erini et al., 2017; Almeida et al., 2019; Zhu Y. et al., 2019; Luo et al., 2021), Ir (Chang et al., 2019; Liang et al., 2019), Sn (Song et al., 2017; Rizo et al., 2018; Roca-Ayats et al., 2018; Wang et al., 2020; Zhu et al., 2020). The synergistic effect and electronic coupling effect are believed to exist in these multicomponent systems, leading to the enhancement in catalytic properties for EOR. Sn, as an oxophilic metal, is one of the most commonly used elements in the design of advanced EOR electrocatalysts. It is widely accepted that the oxophilic metals would offer additional adsorbed hydroxyl groups (OH_ad_) at low potential to promote further oxidation of the carbonaceous intermediates on the Pt sites, thereby alleviating CO poisoning (i.e., bifunctional mechanism) (Song et al., 2017; Roca-Ayats et al., 2018). The electronic coupling between Pt and another metal also changes the adsorption strength and configurations of intermediates on Pt sites by shifting the d-band center of Pt (Dai et al., 2018). Taking the Pt-Sn binary system as an example, the Pt-Sn electrocatalysts have a great adjustability in compositions and structures, thus providing a valuable opportunity to tune the electrochemical performance (Rizo et al., 2017; Wu et al., 2018). In this sense, combining the core-shell structure and alloying strategy should be a feasible and powerful means to boost ethanol oxidation. Unfortunately, for EOR, such works are just a rare sight.

Herein, we report one-pot approach for the synthesis of trimetallic Au@Pt_x_Sn_y_ core-shell nanoparticles (NPs) with tunable composition and structure of Pt-Sn alloyed shells. Impressively, the Au@PtSn core-shell NPs with hexagonal PtSn alloyed shells exhibited the highest mass activity and specific activity toward EOR in alkaline electrolyte, which are 13.0 and 12.7 times higher than those of the commercial Pt/C. We attributed such huge enhancement to the synergistic effect between the core-shell and alloyed surface structures, causing an appropriate d-band center that determined by density functional theory (DFT) calculations.

## Experimental section

### Chemicals

Chloroauric acid tetrahydrate (HAuCl_4_·4H_2_O, Sinopharm, 99.9%), platinum (II) acetylacetonate (Pt (acac)_2_, Sigma-Aldrich, 97%), tin (II) chloride (SnCl_2_, Sigma-Aldrich, 98%), oleylamine (OAm, Aladdin, 80%–90%), oleic acid (OA, Sigma-Aldrich, 90%), cyclohexane (C_6_H_12_, Sinopharm, 99.7%), ethanol (C_2_H_5_OH, Sinopharm, 99.7%), trichloromethane (CHCl_3_, Sinopharm, 99%), carbon black (XC-72R, Cabot), tert-butylamine (C_4_H_11_N, Aladdin, 99%), methanol (CH_3_OH, Sinopharm, 99.5%), isopropyl alcohol ((CH_3_)_2_CHOH, Sigma-Aldrich, 99.7%), Nafion 117 solution (Sigma-Aldrich, 5%), potassium hydroxide (KOH, Sigma-Aldrich, 99.99%), ethanol (C_2_H_5_OH, Macklin, 99.8%, for electrochemical measurements). All the chemicals and materials were used as received.

### Synthesis of Au@Pt, Au@Pt_3_Sn, Au@PtSn, Au@PtSn_2_, Au@PtSn-2 core-shell NPs

In a typical preparation of Au@PtSn NPs, 0.025 mmol HAuCl_4_·4H_2_O, 0.025 mmol Pt (acac)_2_, 0.025 mmol SnCl_2_ were dissolved into a mixture containing 5 ml OAm and 0.25 ml OA in a 20 ml glass vial. The mixture was stirred over 30 min to form a homogeneous solution. Subsequently, the resulting solution was heated to 200°C in an oil bath under vigorous stirring and kept it for 2 h. After naturally cooling to room temperature, the product was collected by centrifugation and washed with cyclohexane and ethanol for three times. For the synthesis of Au@Pt, Au@Pt_3_Sn, Au@PtSn_2_ core-shell NPs, no SnCl_2_, 0.0125 and 0.05 mmol SnCl_2_ were used, respectively. For the synthesis of Au@PtSn-2 core-shell NPs, we double the amount of Pt (acac)_2_ and SnCl_2_ relative to those used in the preparation of the Au@PtSn NPs, with other conditions being the same as the typical procedure.

### Characterizations

The transmission electron microscope (TEM) and high-resolution transmission electron microscope (HRTEM) images were achieved from a Hitachi HT-7700 microscope operated at 100 kV and a FEI Tecnai G2 F20 microscope operated at 200 kV, respectively. A FEI Titan ChemiSTEM equipped with a probe-corrector and a Super-X EDX detector system was employed to obtain the high-angle annular dark-field scanning TEM (HAADF-STEM) and energy dispersive X-ray (EDX) mapping images. The X-ray diffraction (XRD) characterization was performed on a Rigaku Ultima Ⅳ x-ray diffractometer with graphite monochromatized Cu Kα radiation (*λ* = 1.54178 Å). The chemical states of samples were characterized by a Shimadzu AXIS Supra X-ray photoelectron Spectroscopy (XPS) with Al Kα radiation. The percentage of each element in the electrocatalysts was recorded by a Thermofisher iCAP Pro Ⅹ inductively coupled plasma atomic emission spectrometer (ICP-AES).

### Electrochemical measurements

All the electrochemical measurements were conducted in a three-electrode cell using a CHI 760e electrochemical workstation at the room temperature. A saturated calomel electrode (SCE), a Pt wire and a glass-carbon rotating disk electrode (GCE) (diameter: 5 mm and area: 0.196 cm^2^) were used as reference electrode, counter electrode and working electrode, respectively. The as-received data had been converted to reversible hydrogen electrode (RHE). For preparing the working electrode, catalyst ink was needed to be produced. For this purpose, 5 mg catalyst was dispersed in a mixture containing 4 ml deionized water, 1 ml isopropyl alcohol and 0.025 ml 5 wt% Nafion solution, and ultrasonicated for a while to ensure adequate dispersion. After that, a certain volume of ink containing 2 μg noble metals (Pt + Au) was dropped onto the GCE and dried in the air. The cyclic voltammetry (CV) tests were performed in an Ar-saturated 1 M KOH solution between 0 and 1 V vs. RHE at a scan rate of 50 mV/s. The ethanol oxidation reaction (EOR) measurements were conducted in an Ar-saturated 1 M KOH +1 M ethanol (EtOH) solution between 0.37 and 1.37 V vs. RHE at a scan rate of 50 mV/s. The chronoamperometry (I-t) curves were recorded at 0.72 V vs. RHE for 3,600 s in an Ar-saturated 1 M KOH +1 M EtOH solution. The electrochemical active surface area (ECSA) was determined by CO stripping measurements through integrating the oxidation peak of CO in 1 M KOH solution. The mass and specific activities were calculated by normalizing the current density with respect to the mass loading and the ECSA of the catalyst, respectively.

## Results and discussion

### Characterizations of Au@Pt_x_Sn_y_ core-shell nanoparticles

**FIGURE 1:**
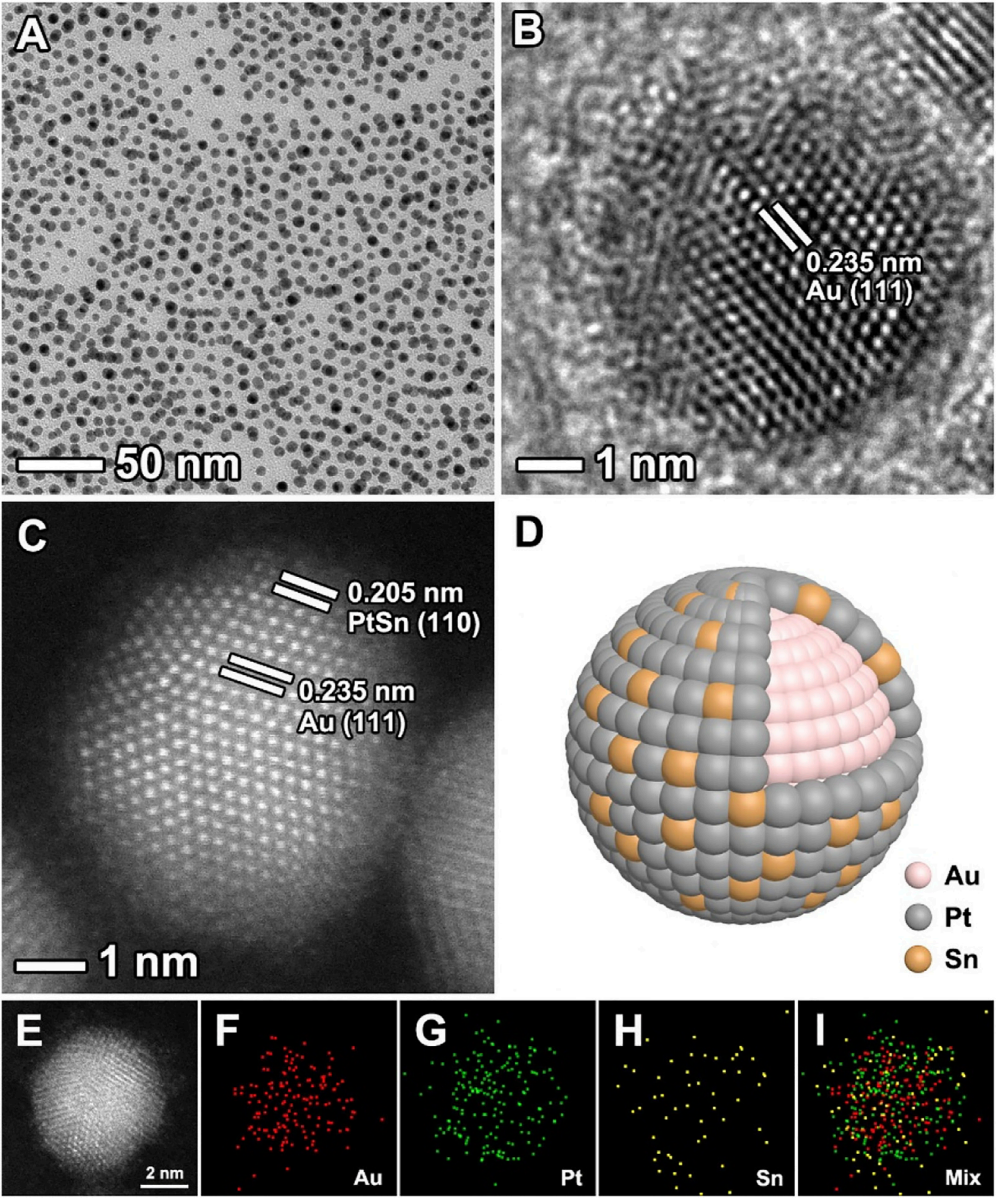
**(A)** TEM image **(B)** HRTEM image **(C)** aberration-corrected HAADF-STEM image **(D)** schematic diagram, and **(E–I)** EDX mapping images of the Au@PtSn core-shell nanoparticles.

The Au@PtSn core-shell NPs were synthesized through a one-pot approach by co-reducing of HAuCl_4_·4H_2_O, Pt (acac)_2_ and SnCl_2_ in a mixture containing oleylamine and oleic acid at 200°C. The morphology, structure and composition characterizations of the Au@PtSn core-shell NPs are shown in Figure 1. As observed, the nanoparticles are well dispersed and have a uniform size distribution with a diameter of 5.51 ± 0.80 nm (Figure 1A and Supplementary Figure S1A). The HRTEM image of an individual nanoparticle in Figure 1B shows that the fringes with a lattice spacing of 0.235 nm can be indexed to the {111} planes of face-centered cubic (*fcc*) Au core. The HAADF-STEM images show the different contrast in the interior and exterior of the nanoparticle, suggesting a core-shell structure (Figures 1C,D). The core-shell structure can also be confirmed by the EDX mapping and line-scan analyses (Figures 1E–I, Supplementary Figure S1B). As observed, the shells are dominated by Pt and Sn, while, Au mainly distributes in the cores, indicating a Au (core)-PtSn (shell) structure. We also synthesized Au@Pt, Au@Pt_3_Sn, Au@PtSn_2_ core-shell nanoparticles by changing the amount of SnCl_2_ precursor, with other conditions being the same as the typical procedure. Through TEM and EDX analyses (Supplementary Figures S2–S4), these samples have similar size and core-shell structure with different compositions compared to the Au@PtSn NPs. The atomic ratios of these three samples were determined by ICP-AES analysis, showing 3:1, 1:1, and 1:2 of Pt/Sn for the Au@Pt_3_Sn, Au@PtSn, and Au@PtSn_2_ core-shell NPs, respectively (Supplementary Table S1).

To further understand the growth mechanism of such core-shell nanostructures, we carried out a set of experiments by collecting the nanoparticles at different reaction times. As shown in Supplementary Figure S5, Au^3+^ ions were preferentially reduced and rapidly formed small nanoparticles. The content of Au in the solution determined by ICP-AES hardly increased anymore after 80 s, indicating that Au^3+^ ions were almost completely reduced. But at this moment, Pt^2+^ and Sn^2+^ ions had not been reduced yet. We found that Pt^2+^ and Sn^2+^ ions were partially reduced after 3 min. As the reaction proceeded, the content of Pt and Sn gradually increased while maintaining an atomic ratio of ∼1. In addition, we conducted a control experiment in which no Au precursor was added. From TEM image in Supplementary Figure S6, the dendritic nanostructures instead of core-shell nanostructures were obtained. This result indicated that the pre-formed Au nanoparticles acted as seeds to promote conformal growth of PtSn shells.

**FIGURE 2:**
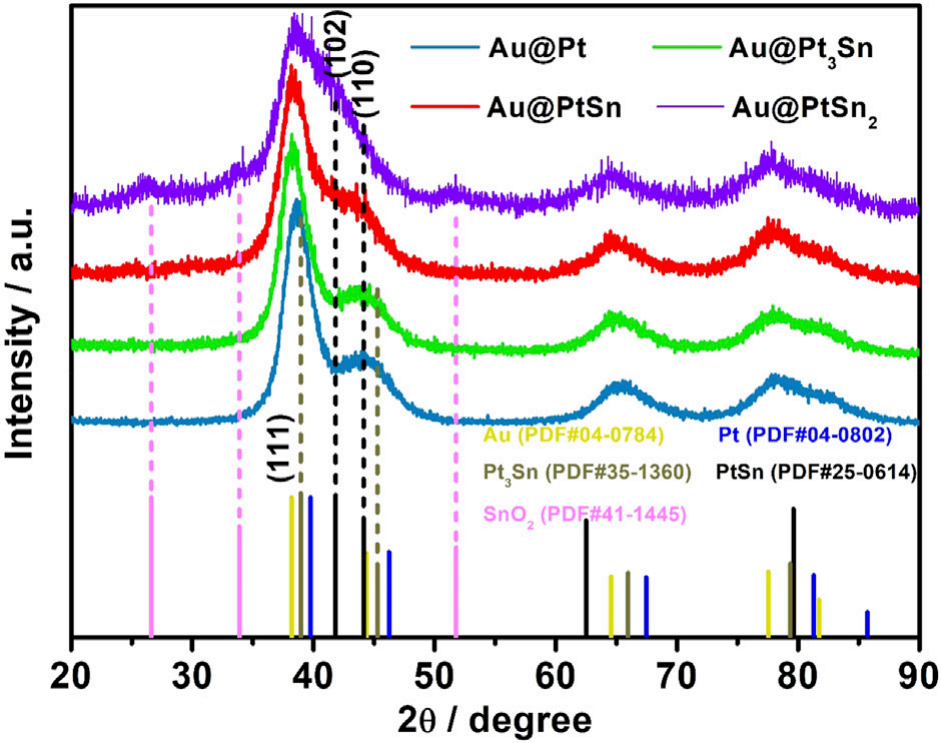
XRD patterns of the Au@Pt_x_Sn_y_ nanoparticles.

The crystal structure of PtSn shells in the core-shell nanostructures with different Pt/Sn atomic ratios was characterized by XRD analysis (Figure 2). As observed, the (111) diffraction peak of the Au@Pt sample is located at 38.7° between those of standard *fcc* Au (111) and *fcc* Pt (111) (i.e., 38.2° vs. 39.8°). The (111) diffraction peak of standard *fcc* Pt_3_Sn is located at 38.9°, which is lower than that of *fcc* Pt (111). As such, the (111) diffraction peak of the Au@Pt_3_Sn sample shifted to a lower angle relative to that of the Au@Pt sample due to the smaller atomic size of Sn than Pt. When the molar ratio of Sn and Pt increased to 1, the (102) diffraction peak associated with hexagonal PtSn phase appeared and the (111) diffraction peak of *fcc* phase shifted to the location of Au. This result demonstrates that the *fcc* Pt_3_Sn phase transforms to the hexagonal PtSn phase with the increase in the amount of Sn precursor fed in the synthesis. As the Sn/Pt molar ratio increased to 2, the (102) diffraction peak of hexagonal PtSn phase is much more obvious. However, the diffraction peaks associated with tin oxide (SnO_2_) appeared, which can be attributed to the oxidation of excess Sn in the presence of residual oxygen (Rizo et al., 2017).

**FIGURE 3:**
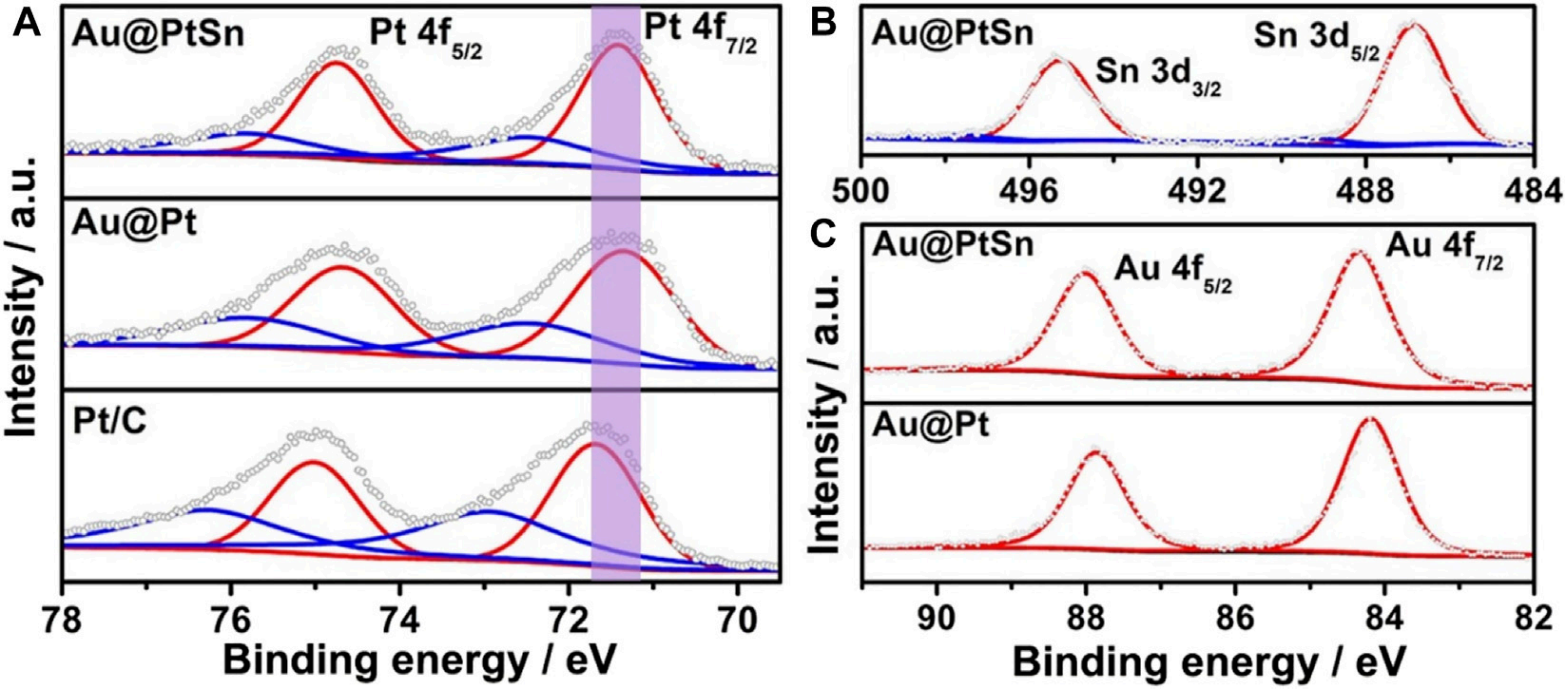
**(A)** Pt 4f **(B)** Sn 3 days and **(C)** Au 4f XPS spectra of the Au@PtSn, Au@Pt and commercial Pt/C, respectively.

The surface compositions and valence states of such core-shell nanostructures were characterized by the XPS measurements. The binding energies of Pt 4f, Sn 3 days and Au 4f were all corrected with respect to that of C 1s peak at 284.5 eV. The fitting curves of Pt 4f and Sn 3 days peaks indicate that Pt and Sn elements on the surface both exist in metallic and oxidation states (Pt^0^/Pt^Ⅱ^, Sn^0^/Sn^Ⅳ^), with the metallic state being in the majority (Figures 3A,B). For Au element, no peak of the oxidation state was observed in Figure 3C, indicating that all Au exists in the metallic state (Zhang et al., 2021; Bi et al., 2020; Zhang T. et al., 2018). Compared to the binding energy of Pt^0^ in the commercial Pt/C, there is an obvious negative shift for that of the Au@Pt NPs (Figure 3A), owing to the tensile strain in the Pt shells and the electronic coupling both introduced by the inner Au cores (Liu et al., 2021). However, for the Au@PtSn NPs, a slight positive shift of the binding energy of Pt^0^ can be observed compared to the Au@Pt NPs (Figure 3A), due to the formation of PtSn alloy. As a result, the binding energy of Pt^0^ for these three samples increased in a sequence of Au@Pt < Au@PtSn < Pt/C (Figure 3A). The XPS spectra of other two samples (Au@Pt_3_Sn and Au@PtSn_2_) were also analyzed, as shown in Supplementary Figure S7. The binding energy of Pt^0^ of these two samples is close to that of the Au@PtSn NPs and is also located between those of the Au@Pt NPs and commercial Pt/C. As such, the core-shell structure with Au as the inner cores and the alloying between Pt and Sn in the shells play critical roles in modulating the electronic structure of surface Pt, which is likely to be beneficial for EOR (He et al., 2021a). In addition, the percentage of the metallic and oxidation state of Sn in Au@Pt_3_Sn, Au@PtSn and Au@PtSn_2_ are 94.3% Sn^0^/5.7% Sn^Ⅳ^, 91.5% Sn^0^/8.5% Sn^Ⅳ^ and 87.9% Sn^0^/12.1% Sn^Ⅳ^, respectively, calculated by the peak area of Sn^0^ and Sn^Ⅳ^ in the XPS spectra (Figure 3B, Supplementary Figure S7C). The much more Sn^Ⅳ^ in the Au@PtSn_2_ agrees well with the appearance of diffraction peaks associated with SnO_2_ in the XRD pattern (Wang et al., 2020; Zhang Z. et al., 2018).

### Electrochemical performances and analysis

**FIGURE 4:**
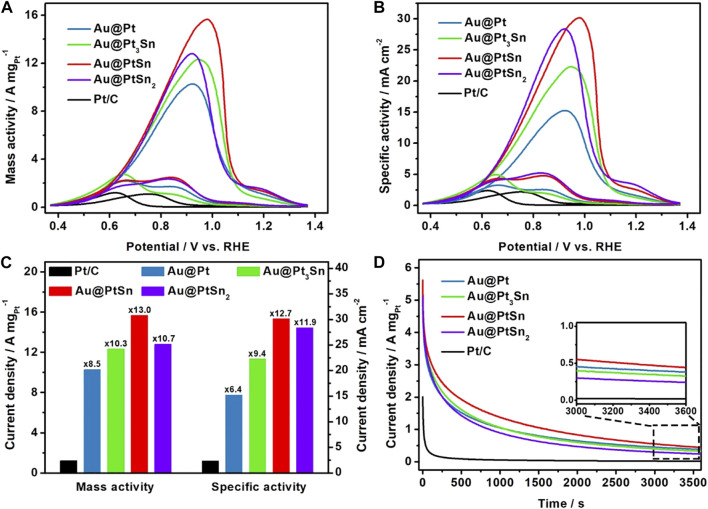
**(A,B)** Cyclic voltammograms (CV) curves normalized by Pt loadings and ECSAs, respectively **(C)** mass and specific activities at the peak position of forward curves and **(D)** current-time (I-t) curves at 0.72 V of the Au@Pt_x_Sn_y_ core-shell NPs and commercial Pt/C in Ar-saturated 1 M KOH +1 M ethanol solution at a scan rate of 50 mV/s.

The Au@Pt, Au@Pt_3_Sn, Au@PtSn, Au@PtSn_2_ core-shell NPs were loaded onto the carbon black (Cabot XC-72R) and then evaluated as electrocatalysts for EOR using a three-electrode system. The cyclic voltammetry (CV) tests of these carbon-supported electrocatalysts were first conducted to clean the surface in Ar-saturated 1 M KOH solution between 0 and 1 V vs RHE at a scan rate of 50 mV/s (Supplementary Figure S8). The currents of CV curves had been normalized against the Pt loading on the GCE. The ECSA of each sample was determined by CO stripping measurements through integrating the oxidation peak of CO in 1 M KOH solution (Xiong et al., 2017) (Supplementary Figures S9A–E). As listed in Supplementary Table S2, the ECSAs of the Au@Pt, Au@Pt_3_Sn, Au@PtSn, Au@PtSn_2_ NPs and commercial Pt/C are about 67.4, 55.3, 51.9, 45.1, and 50.6 m^2^/g_Pt_, respectively. Considering the larger particle sizes but similar or even larger ECSAs of these four core-shell NPs compared to the commercial Pt/C, we believe that the core-shell structure effectively improves the atomic utilization of Pt. The CV curves for EOR recorded in an Ar-saturated 1 M KOH +1 M ethanol (EtOH) solution between 0.37 and 1.37 V vs RHE at a scan rate of 50 mV/s. The current of each curve was normalized against the Pt mass and the ECSA to obtain the mass activity (MA) and specific activity (SA) for EOR, respectively (Figures 4A,B). The forward scan peaks correspond the oxidation process of ethanol to intermediates, and the backward scan peaks correspond the further oxidation of intermediates (Zhang et al., 2019; Peng et al., 2021; Zhang et al., 2021). As observed, the Au@Pt, Au@Pt_3_Sn, Au@PtSn and Au@PtSn_2_ NPs exhibited much higher mass and specific activities compared to the commercial Pt/C. Notably, the Au@PtSn core-shell NPs achieved the highest MA (15.65 A mg_Pt_^−1^) and SA (30.17 mA cm^−2^), which are 13.0 and 12.7 times higher than those of the commercial Pt/C (Figure 4C). As listed in Supplementary Table S3, we summarized the EOR mass activities of recently reported state-of-art Pt-based electrocatalysts in alkaline electrolyte. Intuitively, our Au@PtSn core-shell NPs outperform most of the previously reported Pt-based electrocatalysts towards EOR in alkaline media. Moreover, it is well accepted that the ratio of the peak current of the forward scan peak and backward scan peak reflects the anti-poisoning ability to the intermediates (Fan et al., 2019; Zhang et al., 2019). The Au@PtSn NPs have a much higher ratio (I_f_/I_r_ = 6.34) than other samples (Supplementary Figure S10), indicating that the Au@PtSn NPs possess an excellent anti-poisoning ability. To further confirm the effect of the Au cores on EOR performance, taking the Au@PtSn NPs as a typical sample, we synthesized another sample with a thicker PtSn shell through doubling the amount of Pt and Sn precursors without changing the amount of Au precursor, denoted as Au@PtSn-2. The morphology, composition and structure characterizations of the Au@PtSn-2 NPs (Supplementary Figures S11, S12) indicate the formation of a core-shell structure with a hexagonal PtSn alloy shell. From the Pt 4f XPS spectra of Au@PtSn-2 (Supplementary Figure S13A), the binding energy of Pt^0^ 4f_7/2_ is 71.46 eV, which is close to that of the Au@PtSn NPs (71.41 eV). The atomic ratio of Au, Pt and Sn in the Au@PtSn-2 sample is 18.2/42.4/39.4, determined by ICP-AES, and the atomic ratio of Pt and Sn is approximately 1:1 (Supplementary Table S4). The ECSA of Au@PtSn-2 sample is 52.9 m^2^/g_Pt_ determined by the CO stripping curves in Supplementary Figure S9F. The Au@PtSn-2 NPs exhibited a mass activity of 8.62 A mg/_Pt_ and a specific activity of 15.65 mA/cm^2^, which are 52.9 and 51.9% of those of the Au@PtSn NPs, respectively (Supplementary Figure S14). As we all known, the thicker the shell, the weaker the influence of the core (Yan et al., 2016; Zhao et al., 2016; Zhang et al., 2021; Zhu J. et al., 2019). This result indicates that the inner Au cores play an important role in boosting the EOR performance, owing to the strain effect and electronic coupling effect (Li et al., 2012; Liu et al., 2021).

**FIGURE 5:**
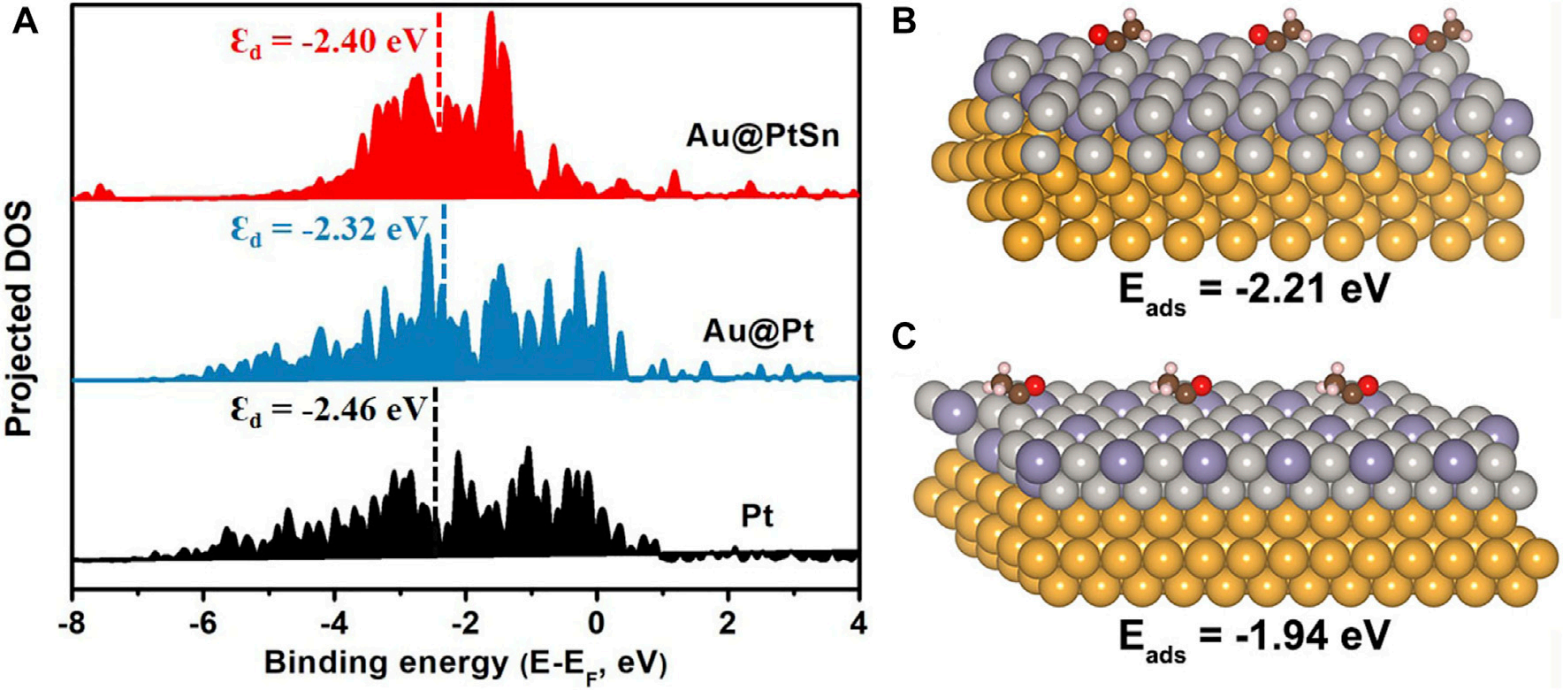
**(A)** PDOS of the Pt 5 days orbits on the surfaces of the Au@PtSn, Au@Pt and Pt models **(B,C)** Adsorption energies of *CH_3_CO on the surfaces of the Au@PtSn and Au@Pt_3_Sn models, respectively.

Generally, the as-synthesized Au@Pt_x_Sn_y_ core-shell NPs exhibited significantly enhanced activities toward alkaline EOR relative to the commercial Pt/C, which can be attributed to the unique core-shell structure with Au as the cores and the formation of Pt-Sn alloy. In addition, the activity of the Au@PtSn NPs is higher than that of the Au@Pt_3_Sn NPs, indicating that the hexagonal PtSn phase is more active than the *fcc* Pt_3_Sn phase towards EOR. For the Au@PtSn_2_ NPs, although the signal of the hexagonal PtSn phase is stronger in the XRD pattern, the existence of SnO_2_ probably covers part of the active sites (smaller ECSA), leading to the decrease of the activity for EOR relative to the Au@PtSn NPs (Du et al., 2014; Fan et al., 2019; Huang et al., 2019). To have a better understanding of the origin of the enhanced activity, DFT calculations were conducted. Nørskov et al. demonstrated that the d-band center of catalysts has a decisive effect on the reactivity and is an important descriptor for designing advanced catalysts (Greeley et al., 2002). An upper d-band center generally means a more reactive surface, which tends to have stronger adsorption of intermediates, while, a surface with a lower d-band center usually has weaker adsorptions of intermediates (Greeley et al., 2002; Li et al., 2012). Since the lattice constant of Au is larger than that of Pt, the introduction of the Au core can expand the lattice of Pt and up-shift the d-band center of Pt. When the Pt shell is alloyed with Sn, the d-band center of Pt is appropriately moved down. Therefore, the d-band center of Au@PtSn falls between those of the Au@Pt and Pt (Figure 5A), resulting in suitable adsorption strengths toward the intermediates. As well known, *CH_3_CO is a key intermediate in the EOR process (Dai et al., 2018), we further calculated the adsorption energies of *CH_3_CO on the Au@Pt_3_Sn and Au@PtSn surfaces. The adsorption energies of *CH_3_CO on Au@PtSn and Au@Pt_3_Sn are −2.21 eV and −1.94 eV, respectively (Figures 5B,C), suggesting that the adsorption of *CH_3_CO on Au@PtSn is stronger than that on Au@Pt_3_Sn. We believe that it is beneficial to promote the subsequent reaction kinetics of *CH_3_CO.

At last, we measured the electrochemical stabilities of all the samples mentioned above by chronoamperometry technique at 0.72 V vs RHE for 3,600 s in an Ar-saturated 1 M KOH +1 M EtOH solution. From the I-t curves in Figure 4D, Supplementary Figure S14F, the Au@PtSn core-shell NPs displayed the best stability among these five samples, with 55.2 and 7.9% of the mass activity left after 1,000 and 3,600 s, respectively. However, the commercial Pt/C only remained 13.2 and 1.0% of the mass activity after 1,000 and 3,600 s, respectively. The superior stability of the Au@PtSn electrocatalyst can be attributed to the excellent anti-poisoning ability towards reaction intermediates. The TEM images of the Au@PtSn electrocatalyst and the commercial Pt/C before and after the stability measurements are shown in Supplementary Figure S15. The decline of the activity of the Au@PtSn electrocatalyst is mainly due to the agglomeration of the NPs and the destruction of the core-shell structure.

## Conclusion

In summary, we reported a facile and one-pot approach to synthesize trimetallic Au@Pt_x_Sn_y_ core-shell NPs with tunable composition and structure of the Pt-Sn alloyed shells. The Au@Pt_x_Sn_y_ core-shell NPs exhibited the substantially enhanced activity and stability for EOR compared to the commercial Pt/C. In addition, the nanoparticles with hexagonal PtSn alloyed shells were superior to those with the *fcc* Pt_3_Sn alloy phase with the Au@PtSn core-shell NPs being the best one. Specifically, the Au@PtSn core-shell NPs achieved the highest mass activity (15.65 A mg_Pt_^−1^) and specific activity (30.17 mA cm^−2^) in alkaline media, which are 13.0 and 12.7 times higher than those of the commercial Pt/C. DFT calculations indicate that the introduction of Au core and alloying Pt with Sn endow Pt with an appropriate d-band center, leading to the huge enhancement in the activity for EOR. This work offers a new sight for the design of high-performance electrocatalysts.

## Data Availability

The original contributions presented in the study are included in the article/Supplementary Material, further inquiries can be directed to the corresponding authors.
