# Supplementary material for: Pt-Sn alloy shells with tunable composition and structure on Au nanoparticles for boosting ethanol oxidation
Source: Front Chem. 2022 Aug 30;10:993894. doi: 10.3389/fchem.2022.993894 (PMC9469013; doi:10.3389/fchem.2022.993894)
Supplement: Supplementary file 1 [file DataSheet1.docx]

Supplementary Material

**Density functional theory (DFT) calculations.**

All the calculations were carried out using spin-polarized density functional theory (DFT) methods using Vienna Ab-initio Simulation Package (VASP)^1,2,3^. The exchange and correlation energies were described by the generalized gradient approximation (GGA) with the Perdew-Burke-Ernzerhof (PBE) functional^4,5^. The projector augmented-wave (PAW) method was used to describe the electron-ion interactions^6,7^. Based on our careful convergence tests, the plane wave energy cutoff was set to 400 eV. The convergence criterion of electronic structure was set to 10^-5^ eV, and the atomic relaxation was continued until the forces acting on atoms were smaller than 0.02 eV/Å. The Brillouin zone was sampled with 3×3×1 Monkhorst-Pack k-point mesh for geometry optimization and 6×6×1 for density of states (DOS) calculation. A Gaussian smearing of 0.05 eV was applied to speed up electronic convergence. The 3-layer 4×4 supercell Au core and 2-layer 2×2 supercell Pt_3_Sn surface were used to build the Au@Pt_3_Sn (111) calculation model. The 3-layer 3×2 supercell Au and 2-layer 3×2 supercell PtSn surface were used to build the Au@PtSn (0001) calculation model. The 3-layer 4×3 supercell Au and 3-layer 4×3 supercell Pt surface were used to build the Au@Pt (111) model. The 4-layer 4×4 supercell Pt were used to build the Pt (111) model. In Au@PtSn, Au@Pt_3_Sn and Au@Pt models, all the Au atoms were fixed, and the other atoms were fully relaxed. In the Pt model, the bottom 2-layer atoms were fixed, and the other atoms were fully relaxed. A vacuum height of 20 Å along the vertical direction was selected to avoid the unwanted interaction between the slab and its period images.

The adsorption energy (E_ad_) of adsorbate on surface is calculated through,

E_ad_ = E_ads-sys_ – E_adsorbate_ - E_surface_

where E_ads-sys_, E_adsorbate_, and E_surface_ are the total energies of the adsorption systems, isolated adsorbate species, and un-adsorbed surface, respectively. The negative E_ad_ denotes an exothermic adsorption process.

The partial density of states of surface Pt atoms were calculated use Au@PtSn, Au@Pt, and Pt models.

**Table S1.** The atomic ratios of Au/Pt/Sn determined by ICP-AES for the samples including Au@Pt, Au@Pt_3_Sn. Au@PtSn and Au@PtSn_2_ core-shell NPs.

| **Samples** | **Atomic ratios of Au/Pt/Sn** |
| --- | --- |
| Au@Pt | 53.8/46.2/- |
| Au@Pt_3_Sn | 40.4/44.9/14.7 |
| Au@PtSn | 30.5/31.5/38 |
| Au@PtSn_2_ | 23.6/26.9/49.5 |

**Table S2.** The ECSAs of the Au@Pt_x_Sn_y_ NPs and commercial Pt/C

| **Samples** | **ECSA / m^2^ g_Pt_^-1^** |
| --- | --- |
| Au@Pt | 67.37 |
| Au@Pt_3_Sn | 55.25 |
| Au@PtSn | 51.88 |
| Au@PtSn_2_ | 45.10 |
| Pt/C | 50.58 |

**Table S3.** EOR mass activities of recently reported state-of-art Pt-based electrocatalysts in alkaline media.

| **Electrocatalysts** | **Mass activities**  **(A mg_Pt_-1)** | **Electrolytes** | **References** |
| --- | --- | --- | --- |
| **Au@PtSn nanoparticles** | **15.65** | **1 M KOH + 1 M EtOH** | **This work** |
| PdH_0.43_@Pt octahedrons | 14.86 | 1 M KOH + 1 M EtOH | Ref. 8 |
| PdPtCu nanorings | 12.42 | 1 M KOH + 1 M EtOH | Ref. 9 |
| Au@PtIr nanoparticles | 58 | 1 M KOH + 1 M EtOH | Ref. 10 |
| PtBi@Pt / graphene | 9.01 | 1 M KOH + 1 M EtOH | Ref. 11 |
| Cu-PtBi nanofiber bundles | 4.00 | 1 M KOH + 0.5 M EtOH | Ref. 12 |
| PtCuNi tetrahedra | 5.6 ± 0.6 | 1 M KOH + 1 M EtOH | Ref. 13 |
| SA In-Pt nanowires | 2.37 | 1 M KOH + 0.5 M EtOH | Ref. 14 |
| SA Ni-Pt nanowires | 5.6 | 1 M KOH + 1 M EtOH | Ref. 15 |
| PtBi@PtRh_1_ nanoplates | 13.02 | 1 M KOH + 1 M EtOH | Ref. 16 |
| PtRhFe nanospheres / MXene | 3.41 | 1 M KOH + 1 M EtOH | Ref. 17 |
| PtRh@SnO_2_ nanowires | 3.16 | 1 M KOH + 1 M EtOH | Ref. 18 |
| Pt / α-PtO_x_ / WO_3_ | 2.76 | 0.1 M KOH + 0.5 M EtOH | Ref. 19 |
| PtCu / Cu_2-x_Se nanowires | 5.03 | 1 M KOH + 1 M EtOH | Ref. 20 |

**Table S4.** The atomic ratio and ECSA of the Au@PtSn-2 core-shell NPs.

| **Sample** | **Atomic ratio of Au/Pt/Sn** | **ECSA / m^2^ g_Pt_^-1^** |
| --- | --- | --- |
| Au@PtSn-2 | 18.2/42.4/39.4 | 52.9 |


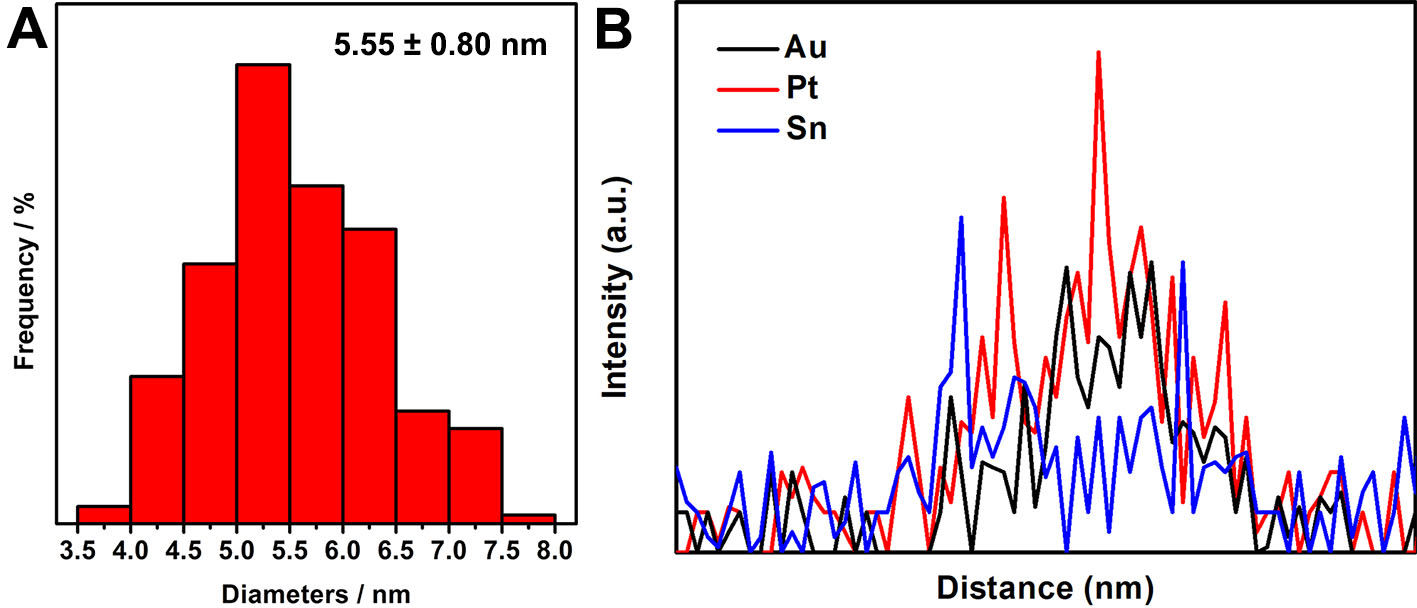


**Figure S1.** (a) The size distribution and (b) EDX line-scan profiles of the Au@PtSn core-shell nanoparticles.


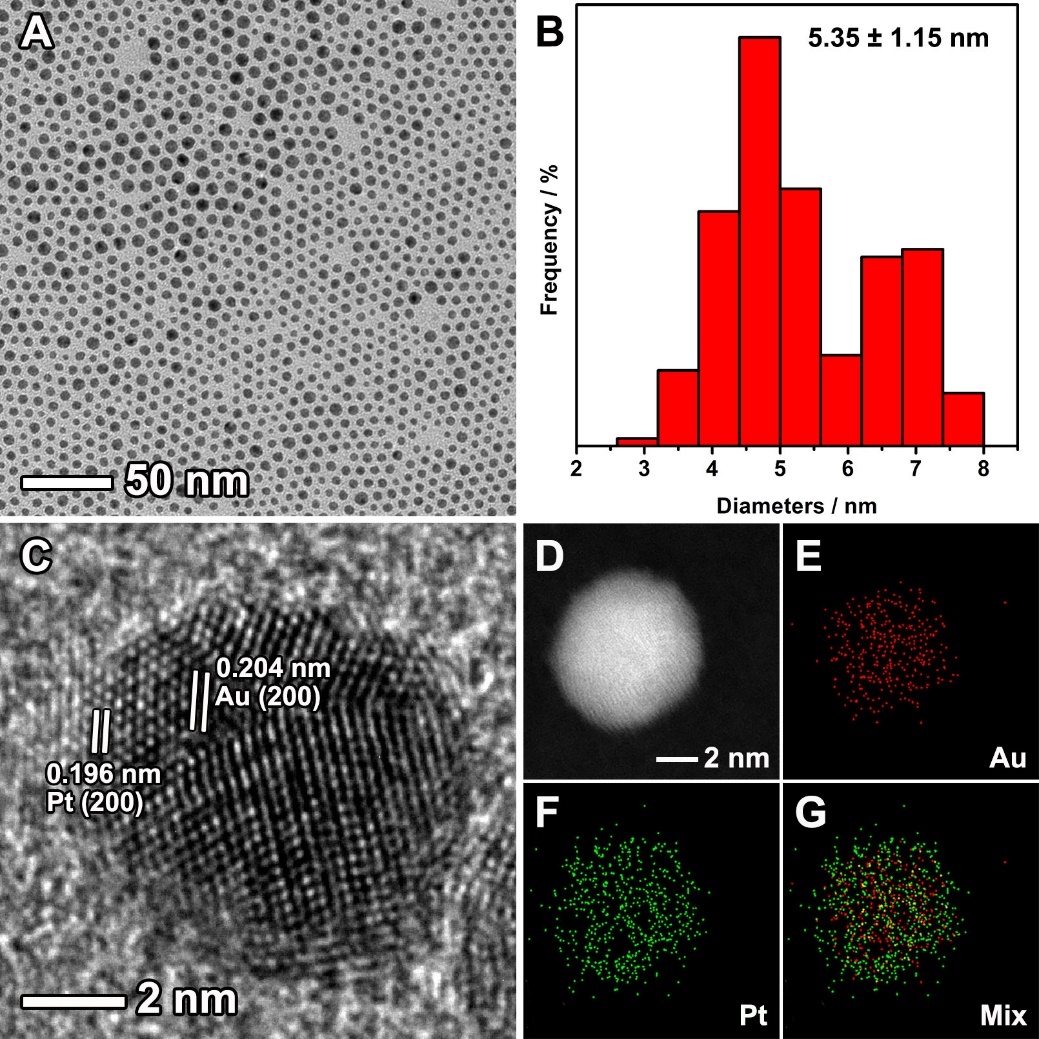


**Figure S2.** (A) TEM image, (B) corresponding size distribution, (C) HRTEM image, (D-G) EDX images of the Au@Pt core-shell NPs.


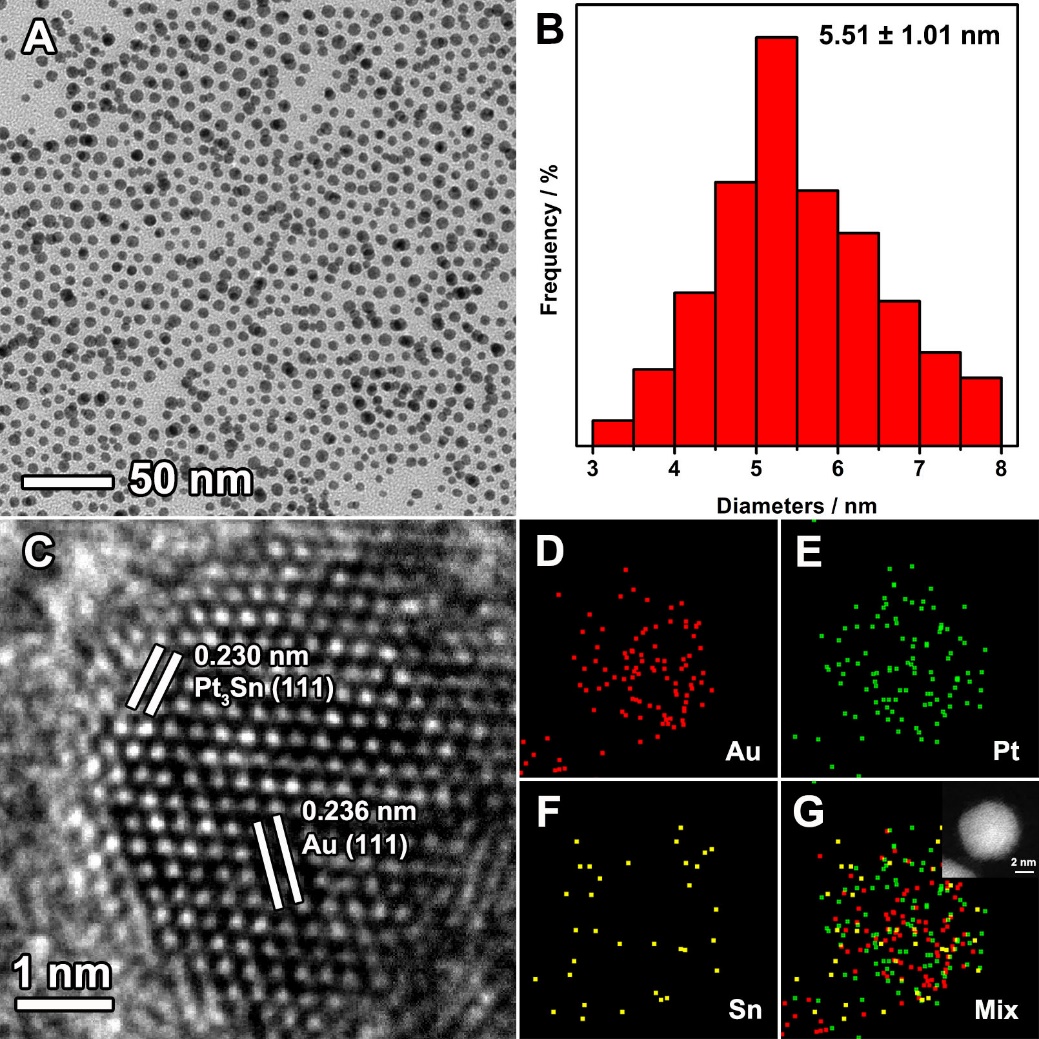


**Figure S3.** (A) TEM images, (B) corresponding size distribution, (C) HRTEM image, (D-G) EDX images of the Au@Pt_3_Sn core-shell NPs.


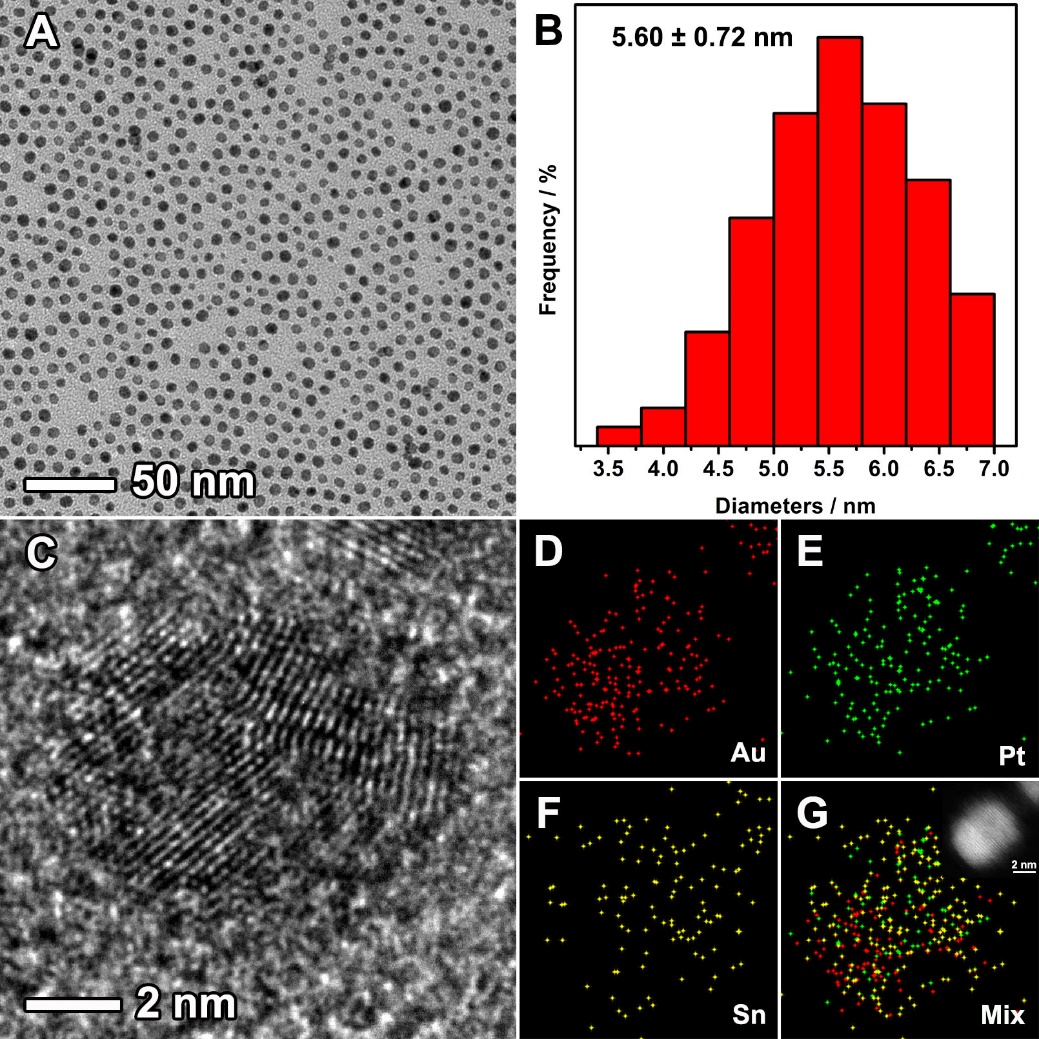


**Figure S4.** (A) TEM image, (B) corresponding size distribution, (C) HRTEM image, (D-G) EDX images of the Au@PtSn_2_ core-shell NPs.


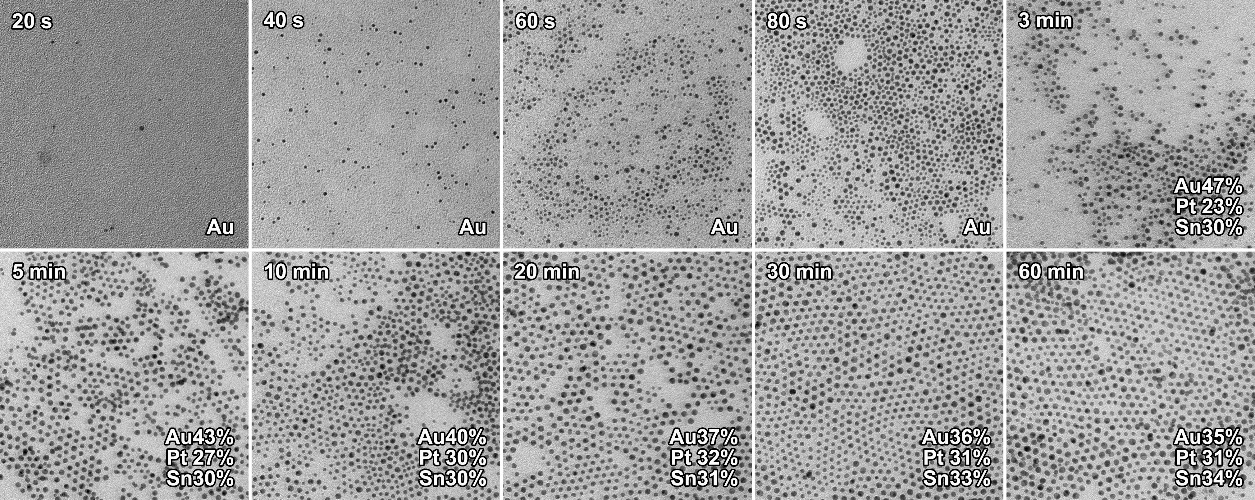


**Figure S5.** TEM images of the nanoparticles collected at different reaction times during the growing process of the Au@PtSn core-shell NPs and the corresponding atomic ratios determined by ICP-AES.


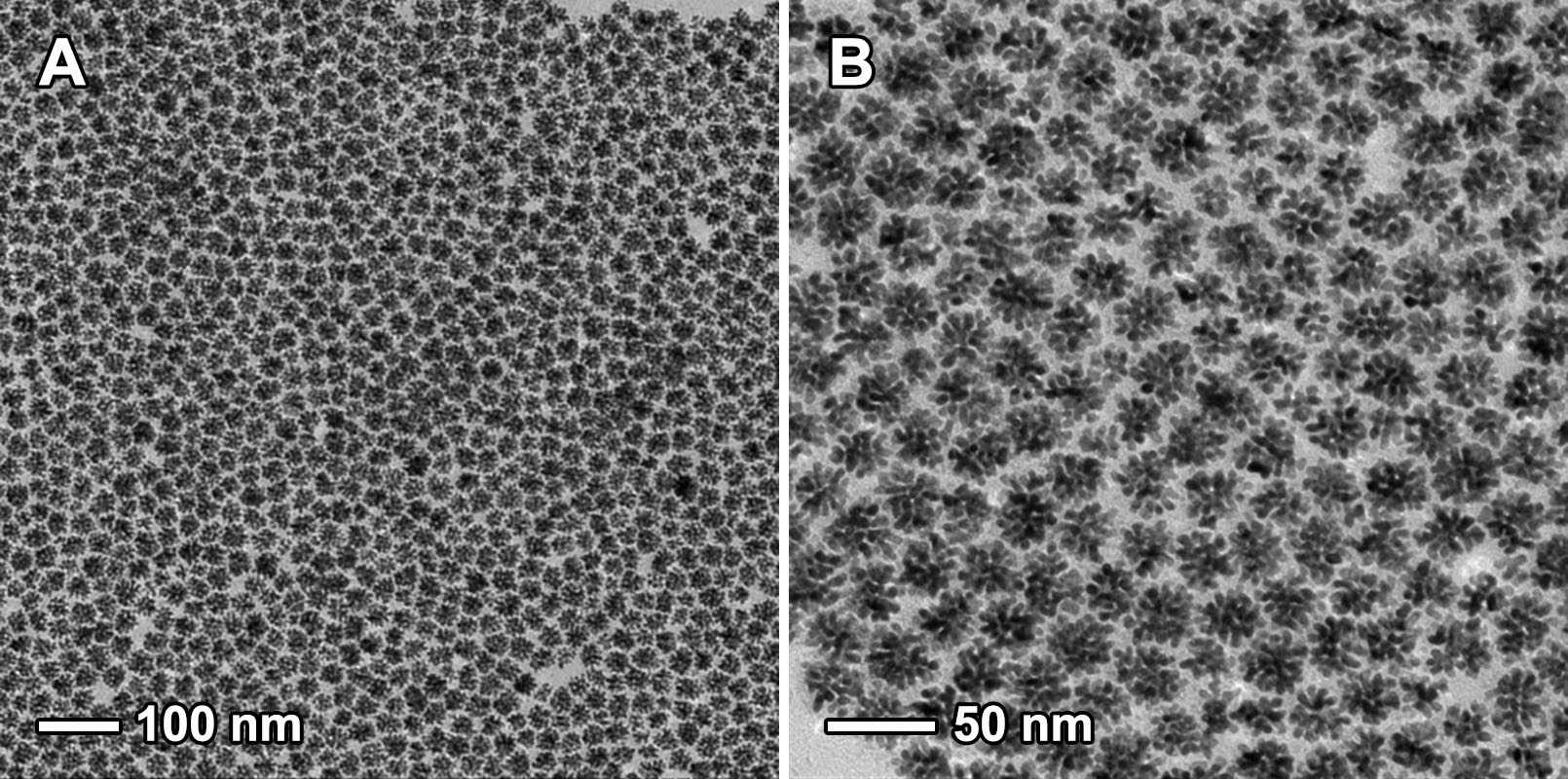


**Figure S6.** TEM images of the nanodendrites prepared using the standard procure except for the HAuCl_4_·4H_2_O precursor.


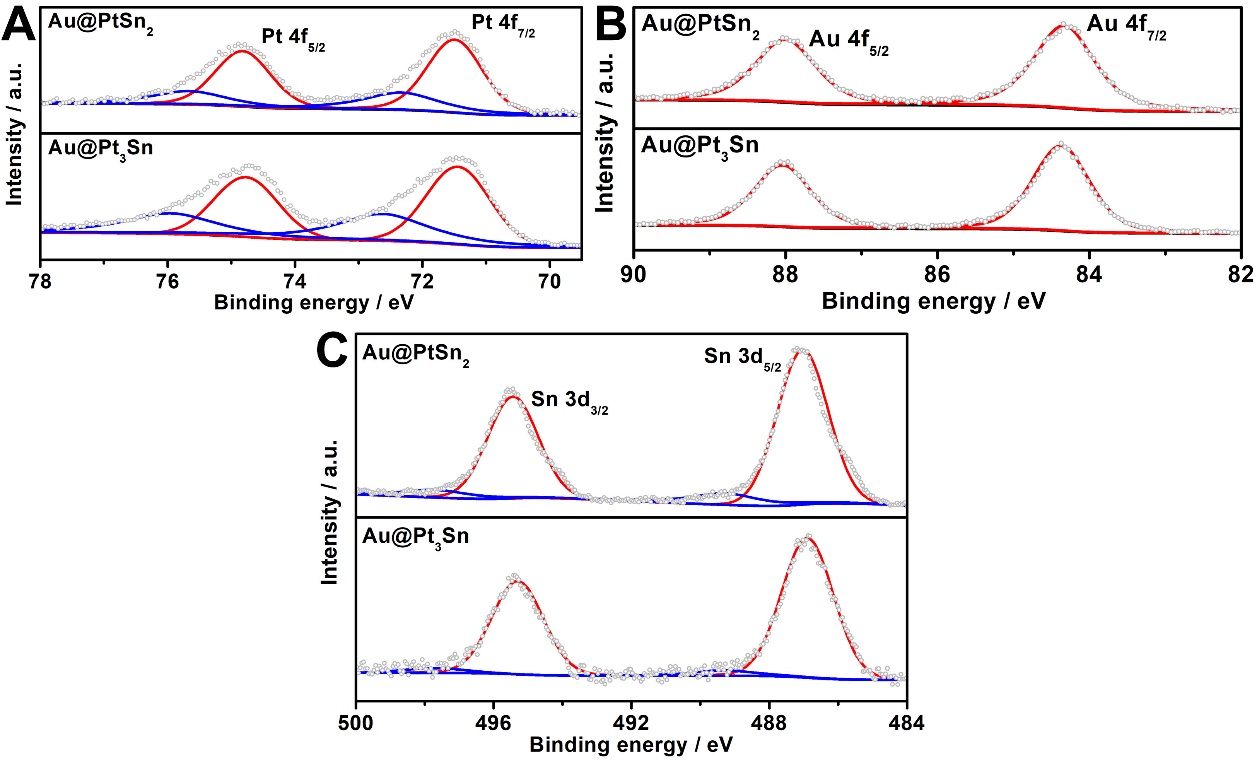


**Figure S7.** (A) Pt 4f (B) Au 4f and (C) Sn 3d XPS spectra of the Au@Pt_3_Sn and Au@PtSn_2_ core-shell NPs, respectively.


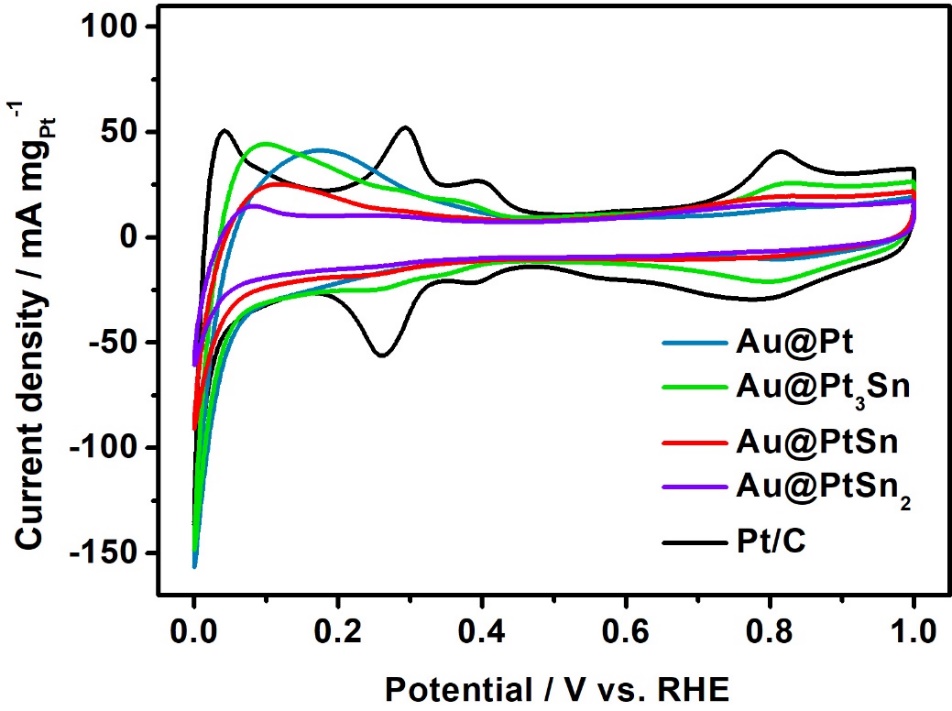


**Figure S8.** Cyclic voltammetry (CV) curves of the Au@Pt_x_Sn_y_ core-shell NPs and commercial Pt/C in Ar-saturated 1 M KOH solution between 0 V and 1 V vs. RHE at a scan rate of 50 mV/s.


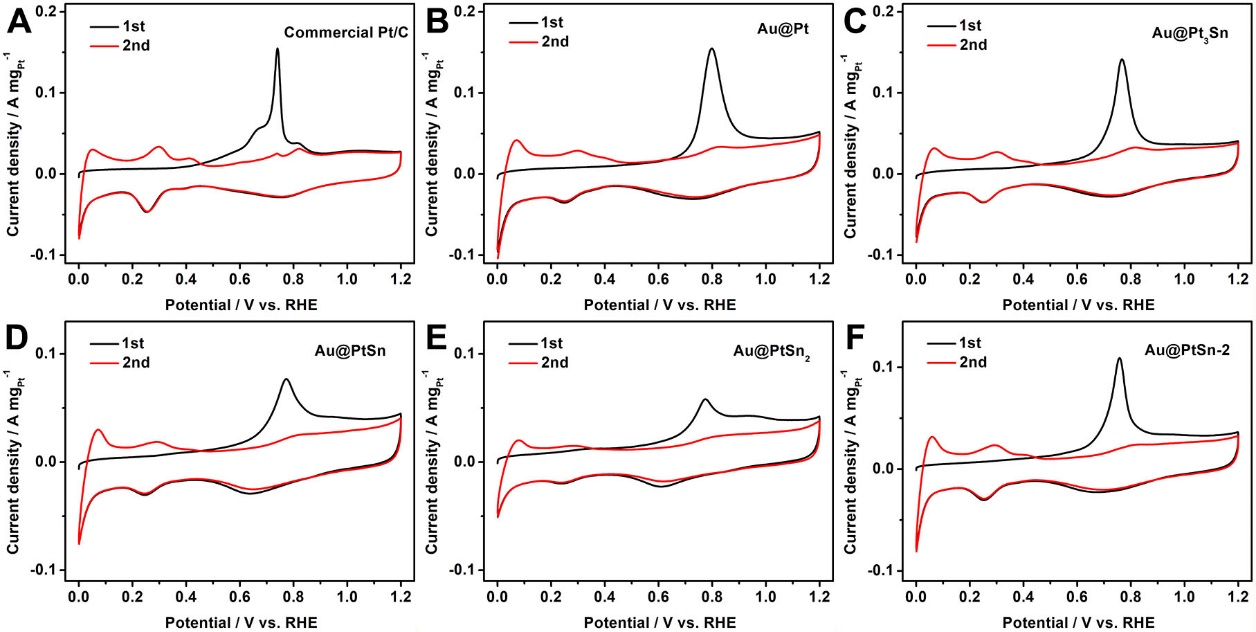


**Figure S9.** CO stripping curves of the six samples recorded in 1 M KOH solution at a scan rate of 50 mV/s.

**
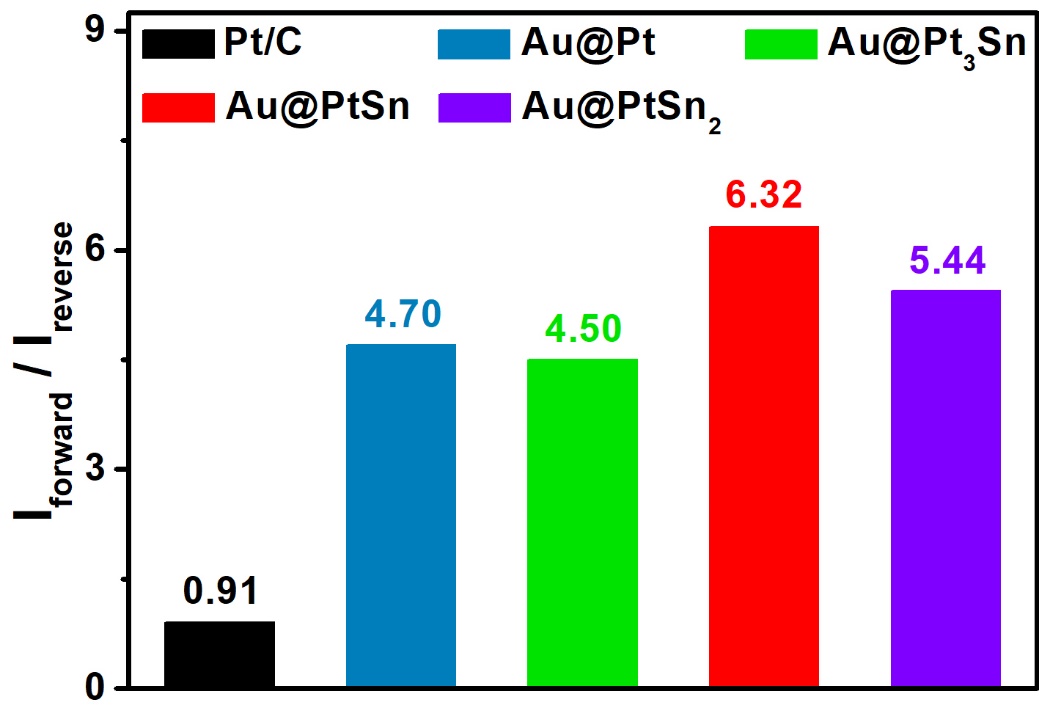
**

**Figure S10.** The ratio of the peak current of the forward scan peak and the backward scan peak (I_f_/I_r_) for the Au@Pt_x_Sn_y_ NPs and commercial Pt/C.


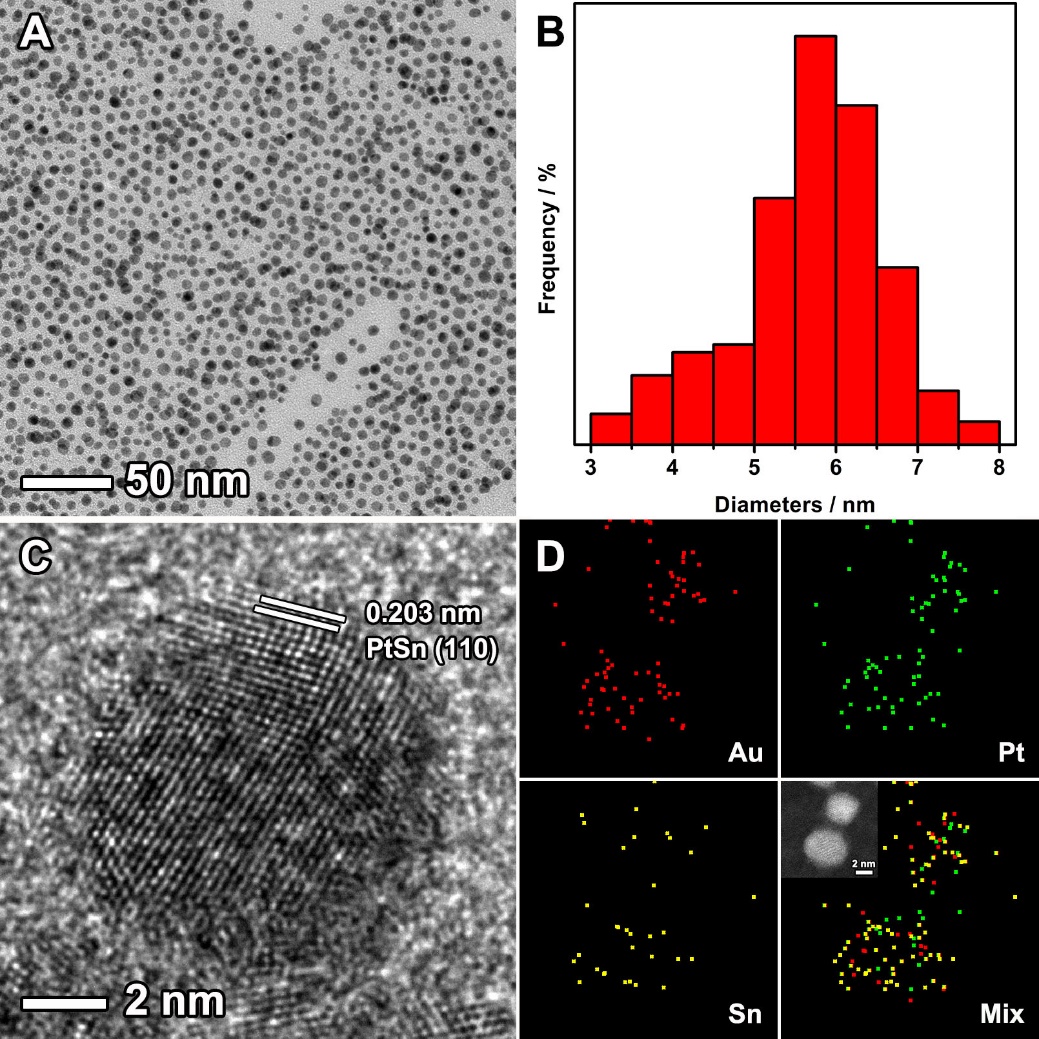


**Figure S11.** (A) TEM image, (B) corresponding size distribution, (C) HRTEM image, (D) EDX images of the Au@PtSn-2 core-shell NPs.


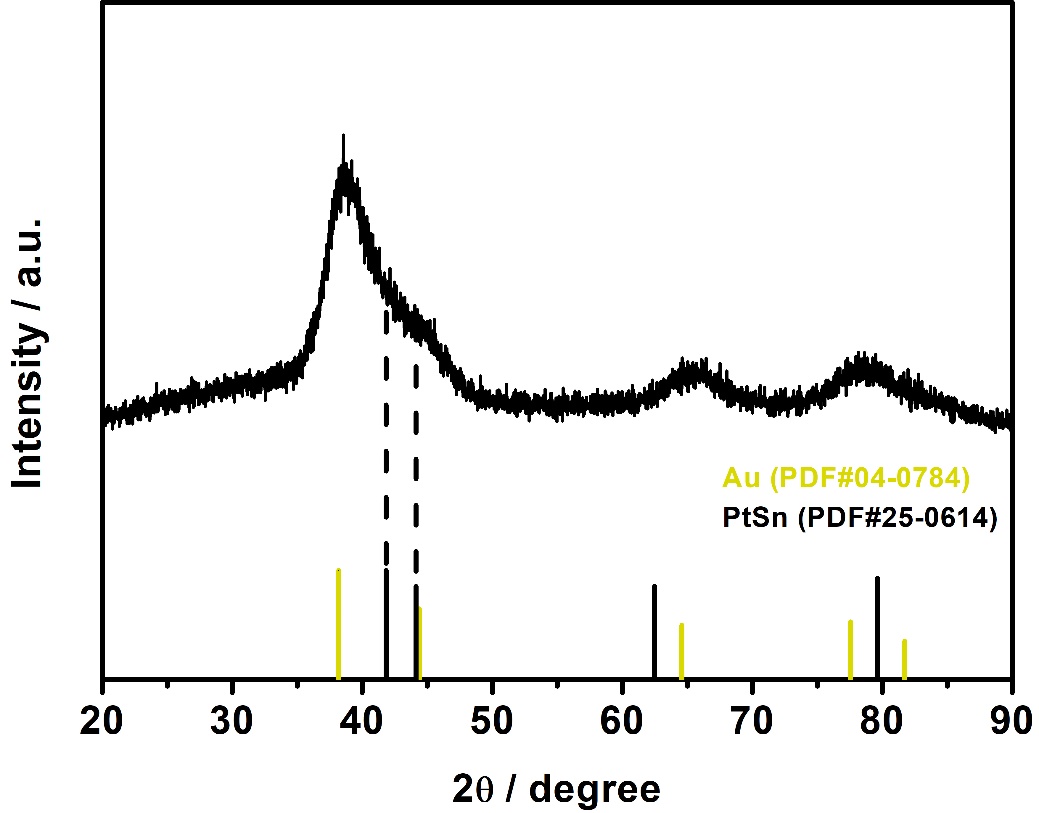


**Figure S12.** XRD pattern of the Au@PtSn-2 core-shell NPs.


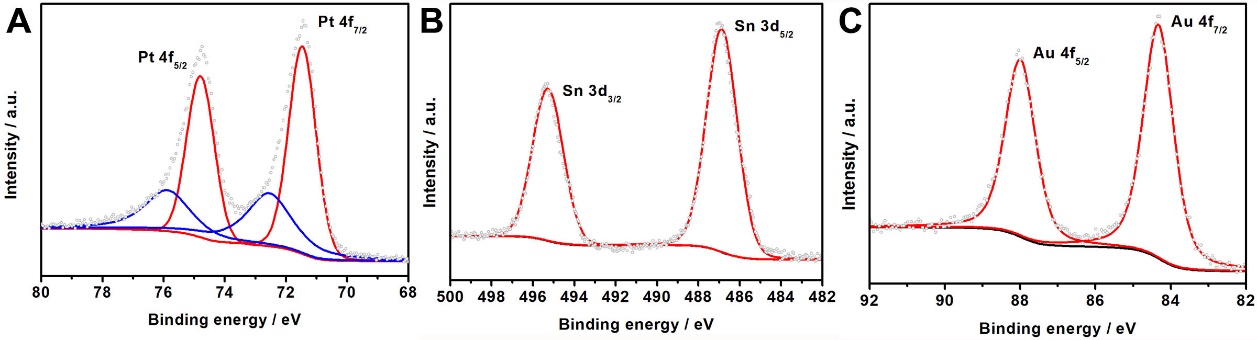


**Figure S13.** (A) Pt 4f (B) Sn 3d and (C) Au 4f XPS spectra of Au@PtSn-2 core-shell NPs.


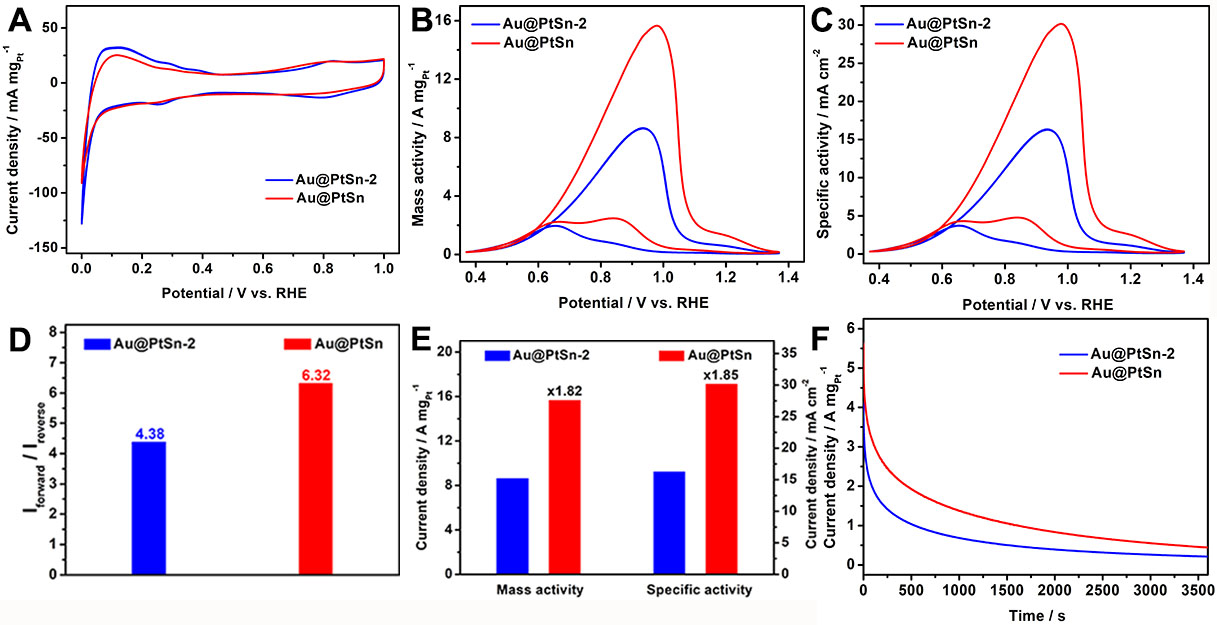


**Figure S14.** (A) CV curves in Ar-saturated 1M KOH solution at a scan rate of 50 mV/s, (B, C) CV curves normalized by Pt loadings and ECSAs in Ar-saturated 1 M KOH + 1 M ethanol solution at a scan rate of 50 mV/s, respectively, (D) I_f_/I_r_, (E) mass and specific activities at the peak position of forward curves, and (F) current-time (I-t) curves at 0.72 V of the Au@PtSn-2 and Au@PtSn core-shell NPs.


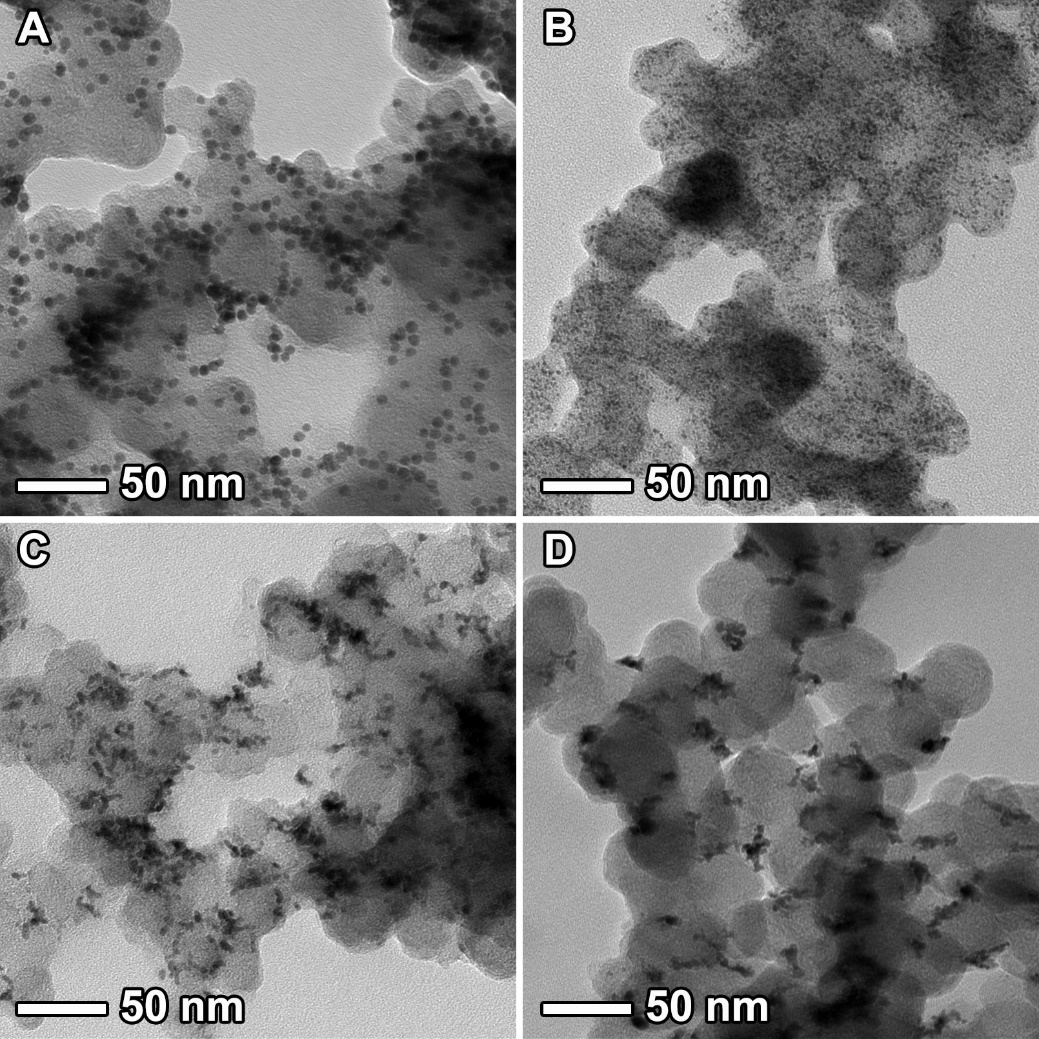


**Figure S15.** TEM images of (A, C) the Au@PtSn/C and (B, D) the commercial Pt/C before and after the stability measurements, respectively.

**References**

1 Kresse, G., Hafner, J. (1993). Ab initio molecular dynamics for liquid metals. Phys. Rev. 47, 558-561.

2 Kresse, G., Furthmüller, J. (1996). Efficient iterative schemes for ab initio total-energy calculations using a plane-wave basis set. Phys. Rev. 54, 11169-11186.

3 Kresse, G., Furthmüller, J. (1996). Efficiency of ab-initio total energy calculations for metals and semiconductors using a plane-wave basis set. Comput. Mater. Sci. 6, 15-50.

4 Perdew, J.P., Wang, Y. (1992). Accurate and simple analytic representation of the electron-gas correlation energy. Phys. Rev. B. 45, 13244-13249.

5 Perdew, J.P., Burke, K., Ernzerhof, M. (1996). Generalized gradient approximation made simple. Phys., Rev. Lett. 77, 3865-3868.

6 Blöchl, P.E. (1994). Projector augmented-wave method. Phys. Rev. B. 50, 17953-17979.

7 Kresse, G., Joubert, D. (1999). From ultrasoft pseudopotentials to the projector augmented-wave method. Phys. Rev. B. 59, 1758-1775.

8 Liu, G., Zhou, W., Ji, Y., Chen, B., Fu, G., Yun, Q., Chen, S., Lin, Y., Yin, P., Cui, X., Liu, J., Meng, F., Zhang, Q., Song, L., Gu, L., Zhang, H. (2021). Hydrogen-intercalation-induced lattice expansion of Pd@Pt core−shell nanoparticles for highly efficient electrocatalytic alcohol oxidation. J. Am. Chem. Soc. 143, 11262-11270.

9 Wang, W., Zhang, X., Zhang, Y., Chen, X., Ye, J., Chen, J., Lyu, Z., Chen, X., Kuang, Q., Xie, S., Xie, Z. (2020). Edge enrichment of ultrathin 2D PdPtCu trimetallic nanostructures effectuates top-ranked ethanol electrooxidation. Nano Lett. 20, 5458-5464.

10 Liang, Z., Song, L., Deng, S., Zhu, Y., Stavitski, E., Adzic, R.R., Chen, J., Wang, J.X. (2019). Direct 12-electron oxidation of ethanol on a ternary Au(core)-PtIr(shell) electrocatalyst. J. Am. Chem. Soc. 141, 9629-9636.

11 Zhang, B., Lai, W., Sheng, T., Qu, X., Wang, Y., Ren, L., Zhang, L., Du, Y., Jiang, Y., Sun, S., Dou, S. (2019). Ordered platinum–bismuth intermetallic clusters with Pt-skin for a highly efficient electrochemical ethanol oxidation reaction. J. Mater. Chem. A. 7, 5214-5220.

12 Zhang, J., Yuan, M., Zhao, T., Wang, W., Huang, H., Cui, K., Liu, Z., Li, S., Li, Z., Zhang, G. (2021). Cu-incorporated PtBi intermetallic nanofiber bundles enhance alcohol oxidation electrocatalysis with high CO tolerance. J. Mater. Chem. A. 9, 20676-20684.

13 Huang, J., Liu, Y., Xu, M., Wan, C., Liu, H., Li, M., Huang, Z., Duan, X., Pan, X., Huang, Y. (2019). PtCuNi tetrahedra catalysts with tailored surfaces for efficient alcohol oxidation. Nano Lett. 19, 5431-5436.

14 Zhu, Y., Zhu, X., Bu, L., Shao, Q., Li, Y., Hu, Z., Chen, C., Pao, C., Yang, S., Huang, X. (2020). Single-atom In-doped subnanometer Pt nanowires for simultaneous hydrogen generation and biomass upgrading. 30, 2004310.

15 Li, M., Duanmu, K., Wan, C., Cheng, T., Zhang, L., Dai, S., Chen, W., Zhao, Z., Li, P., Fei, H., Zhu, Y., Yu, R., Luo, J., Zang, K., Lin, Z., Ding, M., Huang, J., Sun, H., Guo, J., Pan, X., Goddard Ⅲ, W.A., Sautet, P., Huang, Y., Duan, X. (2019). Single-atom tailoring of platinum nanocatalysts for high-performance multifunctional electrocatalysis. Nat. Catal. 2, 495-503.

16 Luo, S., Zhang, L., Liao, Y., Li, L., Yang, Q., Wu, X., Wu, X., He, D., He, C., Chen, W., Wu, Q., Li, M., Hensen, E.J.M., Quan, Z. (2021). A tensile-strained Pt-Rh single-atom alloy remarkably boosts ethanol oxidation. Adv. Mater. 33, 2008508.

17 Wang, P., Cui, H., Wang, C. (2019). In situ formation of porous trimetallic PtRhFe nanospheres decorated on ultrathin MXene nanosheets as highly efficient catalysts for

ethanol oxidation. Nano Energy. 66, 104196.

18 Fan, X., Tang, M., Wu, X., Luo, S., Chen, W., Song, X., Quan, Z. (2019). SnO_2_ patched ultrathin PtRh nanowires as efficient catalysts for ethanol electrooxidation. J. Mater. Chem. A. 7, 27377-27382.

19 Xiao, L., Li, G., Yang, Z., Chen, K., Zhou, R., Liao, H., Xu, Q., Xu, J. (2021). Engineering of amorphous PtO_x_ interface on Pt/WO_3_ nanosheets for ethanol oxidation electrocatalysis. Adv. Funct. Mater. 31, 2100982.

20 Peng, H., Ren, J., Wang, Y., Xiong, Y., Wang, Q., Li, Q., Zhao, X., Zhan, L., Zheng, L., Tang, Y., Lei, Y. (2021). One-stone, two birds: Alloying effect and surface defects induced by Pt on Cu_2-x_Se nanowires to boost C−C bond cleavage for electrocatalytic ethanol oxidation. Nano Energy. 88, 106307.
